# Longitudinal Brain Atrophy Patterns in Early MOG‐Antibody Associated Disease and Relapsing Multiple Sclerosis

**DOI:** 10.1111/ene.70354

**Published:** 2025-11-18

**Authors:** Theodoros Ladopoulos, David Bratek, Carolin Schwake, Ann‐Kathrin Kogel, Barbara Bellenberg, Britta Krieger, Zainab Abbas, Ralf Gold, Carsten Lukas, Ilya Ayzenberg, Ruth Schneider

**Affiliations:** ^1^ Department of Neurology St Josef Hospital, Ruhr University Bochum Bochum Germany; ^2^ Institute for Neuroradiology St Josef Hospital, Ruhr University Bochum Bochum Germany

**Keywords:** brain atrophy, MOGAD, MRI, progression, RRMS, voxel‐based morphometry

## Abstract

**Background:**

Myelin oligodendrocyte glycoprotein antibody‐associated disease can manifest as a relapsing or monophasic condition. Although several MRI studies have shown evident gray and white matter atrophy compared to healthy controls, little is known about regional brain volume dynamics in myelin oligodendrocyte glycoprotein antibody‐associated disease over time.

**Methods:**

In this study, we performed an explorative voxel‐based morphometry to detect brain volumetric differences between myelin oligodendrocyte glycoprotein antibody‐associated disease (*N* = 27), relapsing multiple sclerosis (*N* = 40)—both in early disease stages—and healthy controls (*N* = 45). Furthermore, we investigated the longitudinal brain volume changes over a 2‐year follow‐up period in myelin oligodendrocyte glycoprotein antibody‐associated disease (*N* = 15) and relapsing multiple sclerosis (*N* = 40).

**Results:**

We identified distinct patterns of regional brain volume loss in the patient subgroups compared to healthy controls. In multiple sclerosis patients, bilateral thalamic atrophy was observed, whereas patients with myelin oligodendrocyte glycoprotein antibody‐associated disease showed atrophy of the bilateral fornix and stria terminalis. Our results confirmed longitudinal volume loss in thalamic and infratentorial regions in the relapsing multiple sclerosis group, which was partly related to clinical relapses during the 2‐year follow‐up period. In contrast, no longitudinal gray or white matter changes were found in the myelin oligodendrocyte glycoprotein antibody‐associated disease group.

**Conclusions:**

To our knowledge, this is the first MRI study demonstrating no evidence of regional brain volume loss over time in patients with myelin oligodendrocyte glycoprotein antibody‐associated disease using voxel‐based morphometry, suggesting a different—probably not progressive—pathophysiological background compared to relapsing multiple sclerosis.

**Abbreviations**
DGM: deep gray matter
FLAIR: fluid‐attenuated inversion recovery
GM: gray matter
MOG: myelin oligodendrocyte glycoprotein
MOGAD: myelin oligodendrocyte glycoprotein antibody‐associated disease
MRI: magnetic resonance imaging
MS: multiple sclerosis
NMOSD: neuromyelitis optica spectrum disorder
RRMS: relapsing remitting MS
T1w: T1‐weighted
TIV: total intracranial volume
VBM: voxel‐based morphometry
WM: white matter

## 1 Introduction

Myelin oligodendrocyte glycoprotein (MOG) antibody‐associated disease (MOGAD) is a recently recognized inflammatory demyelinating disorder of the central nervous system (CNS) with distinct clinical phenotypes and poorly understood pathophysiology. Although clinical and imaging manifestations of other demyelinating diseases of the CNS—for example, multiple sclerosis (MS), neuromyelitis optica spectrum disorder (NMOSD)—partly overlap with MOGAD [1], there is evidence for considerable differences in the pathophysiology and natural history of these disease entities [2].

MOGAD has been hypothesized to be a relapsing or rarely monophasic disease. Although evidence in the topic is very sparse, clinical and paraclinical studies do not support the existence of a progressive phase in the course of the disease so far [3, 4, 5]. In contrast, patients with relapsing remitting MS (RRMS) can experience relapse‐related or even relapse‐independent disability progression accompanied by brain atrophy [6]. Longitudinal regional and whole brain atrophy in MS can potentially be influenced by brain lesion load, relapse activity, and disease disability [7, 8]. Regarding aquaporin‐4 antibody‐positive NMOSD, two studies from Japan have reported progression of gray (GM) and white matter (WM) atrophy probably in the context of dying‐back neurodegeneration after optic neuritis or longitudinally extensive myelitis [9, 10].

Voxel‐based morphometry (VBM) is a widely used automated method for characterizing regional gray (GM) and white matter (WM) volume differences between groups of individuals in cross‐sectional or longitudinal settings [11]. Numerous VBM studies reported longitudinal GM and WM atrophy patterns in relapsing and progressive MS subtypes [12, 13]. To our knowledge, no similar longitudinal VBM analyses for regional brain atrophy have been performed in MOGAD to date. In this study, we aimed to perform an exploratory analysis on longitudinal GM and WM atrophy patterns in a single‐center cohort of patients with MOGAD using a voxel‐based method. We compared the temporal dynamics of brain atrophy in MOGAD with a cohort of MS patients and especially investigated the influence of relapse activity on longitudinal brain volume loss. Furthermore, we analyzed cross‐sectional regional volume differences between patients and healthy controls. We have particularly focused on the early stages of both diseases and hypothesized that patients with MOGAD would show no evidence of progressive brain volume loss, in contrast to those with MS.

## 2 Methods

### 2.1 Study Design and Population

We conducted a single‐center retrospective analysis of clinical and MRI data, which were longitudinally acquired between 2013 and 2022 at St. Josef Hospital, Ruhr University Bochum. The data acquisition was conducted specifically for research purposes and was independent of routine clinical care or relapse‐related imaging. 45 healthy controls, 40 patients with RRMS, and 27 patients with MOGAD were recruited and met the international criteria for diagnosis of MS [14] and MOGAD [15, 16]. Individuals with any other intracranial pathologies, incomplete MRI or clinical data, or with a clinical relapse in the last 3 months were excluded. 40 patients with RRMS and 15 patients with MOGAD received a follow‐up examination in a mean time span of 2 years (minimum follow‐up time 12 months, maximum follow‐up time 40 months). The remaining 12 MOGAD patients were lost to follow‐up, primarily due to logistical challenges related to long travel distances to our center, which made participation in follow‐up visits difficult. EDSS scores were acquired by experienced neurologists. Disease duration was defined as the time between first symptoms and baseline MRI scan. Ethics approval was obtained from the Institutional Review Board of the Ruhr‐University Bochum (#15‐5534 and 3714‐10).

### 2.2 MRI Acquisition and Analysis

All MR imaging sequences were performed on a single 3 T MRI scanner (Achieva Philips, Best, Netherlands) using a standardized imaging protocol that included an isotropic 3D fluid attenuated inversion recovery (FLAIR) sequence for lesion quantification (170 sagittal slices, field of view of 240 mm, resolution of 1 × 1 × 1 mm^3^, TR/TE/inversion time in ms of 4800/286/1650, turbo factor of 182, acquisition time of 6 min 30s) as well as a structural isotropic T1‐weighted 3D sequence (3D T1w: T1 fast field echo; 180 sagittal slices; FOV: 240 mm × 240 mm; voxel size: 1 mm × 1 mm × 1 mm; TR, TE, TI/ms: 10/4.6/1000; flip angle: 8°, turbo factor: 164; acquisition time: 6 min) for brain volumetry and voxel‐based morphometry.

MRI data were processed using the Computational Anatomy Toolbox (CAT12 v.12.8, https://www.neuro.uni‐jena.de/cat/) and the Lesion Segmentation Toolbox (LST v.3.0.0, https://www.statistical‐modelling.de/lst.html) for statistical parametric mapping (SPM12, Wellcome Trust Centre for Neuroimaging) implemented in MATLAB R2022b (MathWorks, Natick, MA, USA). Binary lesion maps were created on FLAIR images for patients with MS and MOGAD using the lesion growth algorithm from LST [17] and were manually edited using the FSLeyes image viewer if necessary (https://fsl.fmrib.ox.ac.uk/fsl/fslwiki/FSLeyes). Lesion volumes were quantified, and lesion filling was applied to the 3D T1w image series using LST [17] before brain segmentation and further analyses.

We performed a cross‐sectional comparison between the two patient groups and HC using VBM for GM and WM. All T1‐weighted and FLAIR images were carefully inspected for artifacts before and after preprocessing steps, which included intra‐subject realignment, bias correction, segmentation, and spatial registration. VBM data were subjected to the homogeneity check implemented in CAT12 for quality assurance. GM and WM segmentation images were smoothed with an 8 mm full width half maximum Gaussian kernel and normalized with total intracranial volume (TIV). This kernel size was chosen as it represents a widely accepted compromise between sensitivity and anatomical specificity in group‐level VBM analyses, helping to account for residual inter‐subject anatomical variability. Preprocessing in CAT12 provided volumes of GM, WM, and TIV. We applied a family‐wise error correction for a *p* < 0.05 and an extent threshold of 50 voxels. History of acute demyelinating encephalomyelitis (ADEM) or tumefactive lesions was used as a covariate in our statistical model as a variable possibly affecting WM atrophy in MOGAD compared to HC. For longitudinal analysis, we used the dedicated VBM workflow for longitudinal models for small volume changes implemented in CAT12, which includes the creation of an unbiased midpoint image between baseline and follow‐up, correction for bias field inhomogeneities, segmentation, and high‐dimensional warping to MNI space. This model is optimized for detecting small volume changes over time. GM and WM maps were again modulated and smoothed with an 8 mm FWHM kernel. Furthermore, to test the influence of clinical relapses between baseline and follow‐up MRI examinations on the longitudinal atrophy patterns, we also added this information as a covariate in our analysis. We used the neuromorphometrics (http://neuromorphometrics.com/) and the Mori [18] atlas to determine the anatomical location of the significant clusters in gray and white matter, respectively.

## 3 Statistical Analysis

SPSS software (IBM Corp. Released 2016. IBM SPSS Statistics for Windows, Version 26.0. Armonk, NY: IBM Corp.) was used for the statistical analyses. GM and WM volumes were normalized for physiological body‐size effects by dividing their values by the corresponding subject's intracranial volume (TIV) and multiplying the result by the mean TIV of our study population. To assess the distribution of data in the patient and healthy control (HC) groups, the Shapiro–Wilk test was applied. Most parameters, including EDSS, lesion volume, and WM and GM volumes, did not follow a normal distribution. Therefore, non‐parametric tests (Mann–Whitney *U* and Kruskal–Wallis tests), along with pairwise post hoc tests for multiple comparisons, were used for group comparisons. Results of pairwise comparisons were corrected using the Dunn–Bonferroni method (Table 1: age, TIV, WM, GM, DGM). Effect size coefficients r for all pairwise comparisons using the Mann–Whitney *U* test and *η* ^2^ for all multi‐group comparisons assessed via the Kruskal–Wallis test were calculated [19].

**TABLE 1:** Demographics and global brain MRI volumetry.

Median (IQR)	HC	RRMS	MOGAD	Kruskal–Wallis test	Effect‐size measures
Number (BS/FU)	(45/0)	(40/40)	(27/15)		
Female %*	57.8%	55%	66.7%	n.s.^a^	*η* ^2^ = 0.0004
Age (years)**	31 (27–37)	34 (26–40)	28 (21–46)	n.s.	*η* ^2^ ≈ 0
Disease duration (years)	—	2 (1–3)	2 (1–5)	n.s.^b^	*r* = −0.076
Patients on DMDs, N (%)		31 (77.5%)	19 (70.4%)	n.s.^a^	*r* = −0.074
Time to follow‐up (months)	—	25 (23–26)	25 (23–35)	n.s.^b^	*r* = −0.096
Patients with relapses between BS and FU (%)		8 (20%)	4 (26.7%)	n.s.^a^	*r* = −0.1905
EDSS (BS)	—	1.5 (1.0–2.5)	2.0 (1.0–2.0)	n.s.^b^	*r* = −0.0023
EDSS (FU)	—	1.5 (1.0–2.5)	2.0 (1.0–2.5)	n.s.^b^	*r* = −0.0043
TIV (mL)	1481 (1367.4–1598.9)	1496.7 (1375.8–1605)	1399 (1314.8–1535.3)	n.s.	*η* ^2^ ≈ 0
WM (mL)^c^	519.8 (500.1–531.5)	515.7 (491.6–530.4)	514.2 (486.1–523.6)	n.s.	*η* ^2^ ≈ 0
GM (mL)^c^	670.4 (648.9–691.9)	646.2 (620.4–692.2)	679.8 (637.9–707.2)	n.s.	*η* ^2^ = 0.0076
DGM (mL)^c^	29.8 (28.9–30.67)	28.1 (26.5–30.4)	28.9 (26.8–30.3)	*p* = 0.007	*η* ^2^ = 0.0694
Lesion volume (mL)	—	1.7 (0.7–3.8)	0.1 (0–0.54)	*p* < 0.001^b^	*r* = −0.574

*Note:* Clinical and MRI data refer to the baseline visit unless otherwise specified. The interpretation of effect sizes followed conventional thresholds (small and large effect size for |*r*|: 0.10 and 0.50 respectively, small and large effect size for *η* ^2^: 0.01 and 0.14 respectively).

Abbreviations: BS, baseline; FU, follow‐up; IQR, interquartile range; *N* number.

^a^ Chi‐square test.

^b^ Mann–Whitney *U* test.

^c^ Corrected for TIV.

## 4 Results

**FIGURE 1:**
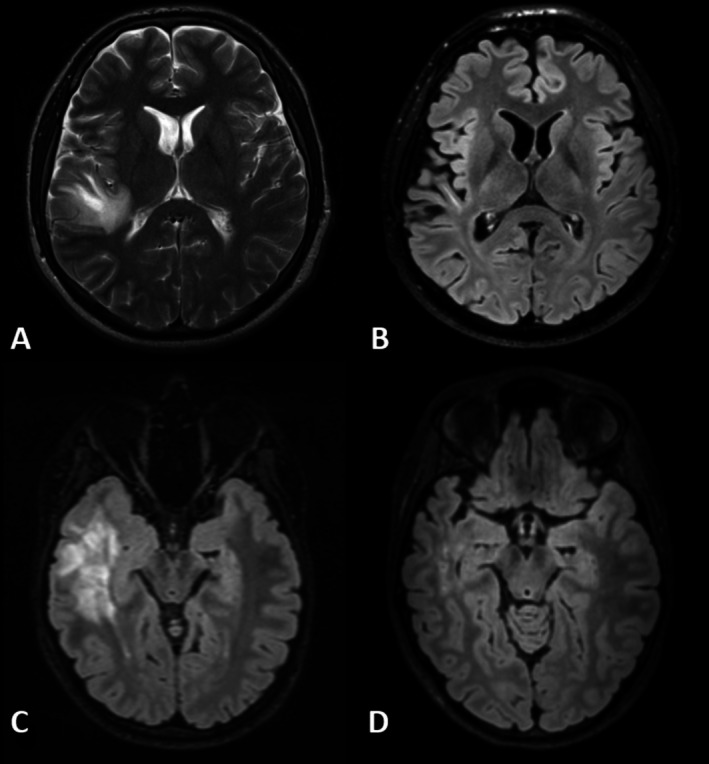
(A) Patient 1 (male, 20 years‐old) with MOGAD and ADEM manifestation in right temporal and parietal lobe (6 years before baseline MRI). (B) Follow‐up MRI scan of patient 1 in the context of our study (6 years later) demonstrating marked reduction of the tumefactive lesion. (C) Patient 2 (female, 19 years old) with MOGAD and ADEM manifestation in right temporal lobe. (D) Follow‐up MRI scan of patient 2 within our study (6 months later) showing almost complete regression of the lesion.

Demographic, clinical, and MRI data are summarized in Table 1. Most of our patients were under immunosuppressive or immunomodulatory treatment (Table S1). Our groups did not significantly differ in terms of sex, age, disease duration, time to follow‐up, incidence of relapses between visits, EDSS, TIV, and WM volume. Although we identified a trend for a lower GM volume in our MS group, the results did not reach statistical significance. Deep gray matter (DGM) volume was significantly lower in the MS and the MOGAD group compared to HC (*p* = 0.007). Whole brain lesion volume in MS patients was higher in comparison with the MOGAD group (*p* < 0.001). The clinical phenotypes of our MOGAD cohort are reported in Table 2. A history of ADEM or tumefactive lesions in temporal lobes was noticed in 7 patients with MOGAD in our cohort (examples are depicted in Figure 1).

**TABLE 2:** Clinical phenotypes of the MOGAD group.

MOGAD – leading clinical phenotypes	Number of patients (%)	Relapses between BS and FU
All	27 (100%)	4
Optic neuritis	11 (40.7%)	2
Bilateral Optic neuritis	7 (25.9%)	1
Myelitis	9 (33.3%)	1
Longitudinal extensive myelitis	5 (18.5%)	1
ADEM or tumefactive lesions (> 2 cm)	7 (25.9%)	1
Monophasic	8 (29,6%)	0

Abbreviations: ADEM, acute demyelinating encephalomyelitis; BS, baseline; FU, follow‐up.

### 4.1 Cross‐Sectional VBM Results at Baseline—Differences Between Groups

**FIGURE 2:**
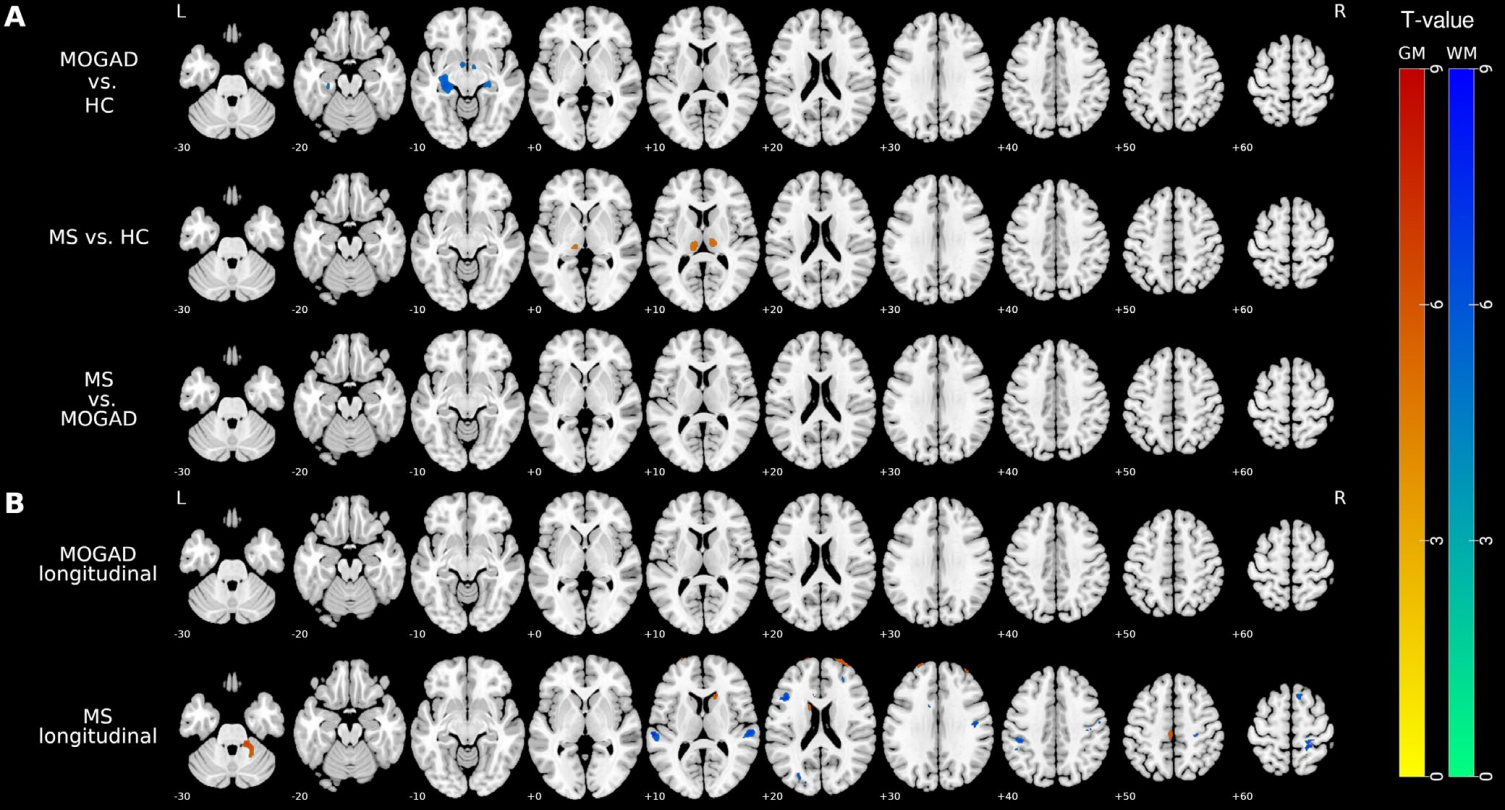
VBM results of GM and WM volume differences in MOGAD, MS, and healthy controls. (A) Cross‐sectional VBM comparisons show significant WM atrophy in MOGAD, involving the fornix, white matter adjacent to the hippocampus, and stria terminalis, and GM atrophy in MS, in the bilateral thalamus. (B) Longitudinal VBM analysis reveals no evidence of significant GM or WM atrophy over time in MOGAD, whereas patients with MS show progressive volume loss in characteristic GM and WM regions. Statistically significant clusters are color‐coded as follows: Red/yellow for GM and blue/green for WM. All results are thresholded at *p* < 0.05 (FWE‐corrected) with a cluster extent threshold > 50 voxels.

We compared baseline MRIs of the HC group with our MS and MOGAD cohorts using VBM analysis for GM and WM. The MOGAD group demonstrated significant WM atrophy (WM adjacent to hippocampus, bilateral fornix and stria terminalis) when compared to HC. No statistically significant GM volume differences could be detected between MOGAD and HC. When the presence of ADEM or tumefactive lesions was added as a covariate in our statistical model, no WM volume loss could be demonstrated in MOGAD compared with HC. In patients with MS, prominent GM atrophy in bilateral thalami was detected compared to healthy individuals, whereas no significant differences for WM were observed. No significant GM and WM volume differences were observed between MOGAD and MS patients. Our results are depicted in detail on Figure 2. Exact coordinates, cluster size, and overlap of the significant clusters with brain regions are shown on Table 3.

**TABLE 3:** Significant VBM clusters for cross‐sectional group comparisons and longitudinal analyses.

Brain region	MNI coordinates			Cluster size (voxels)	% of cluster
	x	y	z		
HC > MOGAD WM					
Right fornix stria terminalis	24	−24	−14	93	32%
Right hippocampus	24	−24	−14	93	20%
Left fornix stria terminalis	−28	−28	−21	842	25%
Left Hippocampus	−28	−28	−21	842	22%
HC > MS GM					
Right thalamus proper	14	−22	6	306	100%
Left thalamus proper	−12	−26	2	407	100%
MS longitudinal TP1 > TP2 GM					
Left cerebellum exterior	−18	−76	−58	60	68%
Left precentral gyrus medial segment^a^	−3	−24	45	103	96%
Left caudate	−14	24	−2	105	66%
Left superior frontal gyrus	−27	68	9	193	65%
Left frontal pole	−27	68	9	193	31%
Right cerebellum exterior^a^	27	−34	−39	260	50%
Right caudate	16	24	4	98	54%
Right superior frontal gyrus^a^	24	70	12	297	44%
Right middle frontal gyrus^a^	24	70	12	297	25%
Right frontal pole^a^	24	70	12	297	26%
MS longitudinal TP1 > TP2 WM					
Left cingulate and cingulum gyrus	−8	21	20	53	96%
Right postcentral gyrus	27	−28	50	70	84%
Right middle frontal gyrus	26	45	14	94	96%
Right superior frontal gyrus	14	27	54	96	100%
Left superior temporal gyrus^a^	−57	−22	6	120	100%
Left middle occipital gyrus	−21	−88	12	144	74%
Left superior occipital gyrus	−21	−88	12	144	24%
Left frontal gyrus	−40	32	12	182	68%
Left middle frontal gyrus	−40	32	12	182	32%
Right superior temporal gyrus^a^	58	−16	4	189	100%
Left supramarginal gyrus^a^	−42	−33	34	225	87%
Right postcentral gyrus^a^	26	−34	52	226	79%
Right superior parietal lobule	26	−34	52	226	21%

*Note:* The neuromorphometrics and the Mori atlas were used for localization in GM and WM, respectively.

^a^ Clusters that remained significant after applying the occurrence of clinical relapses between BS and FU as a covariate in the longitudinal analysis.

### 4.2 Patient Groups—Longitudinal VBM Results

Median time to follow‐up was 25 months for both groups. The MOGAD baseline and follow‐up group did not differ significantly in terms of sex, age, disease duration, and EDSS. Four MOGAD and eight MS patients suffered a clinical relapse between baseline and follow‐up examination. We assessed longitudinal GM and WM volume loss in each patient group separately. No longitudinal WM and GM changes were shown in the MOGAD group.

MS patients exhibited statistically significant clusters of GM atrophy in DGM (bilateral caudate nuclei), infratentorially (cerebellar cortex), in superior and middle frontal gyrus, and in frontal pole. Regarding WM volume loss in MS patients across time, several clusters could also be detected in adjacent WM structures of cingulate and postcentral gyrus, as well as in frontal, temporal, and occipital gyri. Cluster size and overlap with brain regions are summarized in Table S4.

We assessed the potential influence of clinical relapses by including the occurrence of relapses between the baseline and follow‐up scans as a covariate in our longitudinal analyses. No changes were observed in the MOGAD group. In the MS group, following clusters of GM volume loss remained significant: left precentral gyrus (medial segment), right cerebellum exterior, right superior and middle frontal gyrus, and right frontal pole. Regarding the cluster of WM volume loss, left and right superior temporal gyrus, left supramarginal gyrus, and right postcentral gyrus survived the covariate adjustment. The detailed VBM results for MS patients are shown in Table 3 and in Figure S1.

## 5 Discussion

This exploratory VBM study investigated differences in longitudinal brain volume dynamics in patients with early MOGAD and RRMS over a 2‐year time span and additionally provided regional GM and WM atrophy patterns in comparison with HC. We were able to confirm longitudinal volume decrease in GM and WM in MS patients even in the early disease phase as previously described [12, 20]. No evidence of regional brain volume loss could be demonstrated over the course of 2 years in our MOGAD cohort, indicating no evidence of subclinical progression in the context of neurodegeneration in our study group.

Our patient and HC groups showed no between‐group differences for age, sex, disease duration, time to follow‐up, TIV, and disease‐related disability as expressed by EDSS. With a median disease duration of 2 years, we can conclude that the study focuses on early disease stages. The majority of patients with MS or MOGAD were on effective disease‐modifying treatments with a relatively low relapse rate. With a median age of < 35 years, we considered brain atrophy due to age‐related physiological processes in our longitudinal analysis to be subordinate [21]. With regard to global brain volumes, no differences regarding WM volume were exhibited between the patient subgroups and HC, which could possibly be explained by the short disease duration, low lesion load, and EDSS in both patient groups. MS patients showed reduced median total GM and DGM volume compared to MOGAD and HC; however, the results for total GM volume did not reach statistical significance. Particularly for DGM, this could possibly be attributed to the relatively small patient groups (medium effect size) and early phase of the disease compared with other studies in the field. Brain lesion load was higher in patients with RRMS in comparison with MOGAD.

In our cross‐sectional voxel‐based analysis between patients with MOGAD and HC, no differences were observed regarding GM volumes, whereas bilateral fornix and stria terminalis showed significant WM volume loss compared to HC. Fornix is a major hippocampal output tract [22]. As a considerable amount of patients in our MOGAD group had a history of tumefactive lesions or ADEM with temporal lobe and hippocampal involvement, this result could be interpreted as a consequence of Wallerian degeneration. Including the presence of ADEM as a covariate in our analysis allowed us to support this hypothesis, as all of the WM volume differences were removed. In contrast to our findings, recent MRI studies using VBM and surface‐based morphometry (SBM) could demonstrate significant deep gray matter and cortical atrophy in MOGAD compared to healthy controls, especially in pericalcarine, orbitofrontal, and temporal lobe GM [23, 24]. These discrepancies could possibly lie in different image analysis methods (VBM vs. SBM), application of the new diagnostic criteria in our study, younger age (28 vs. 40.9 years) and longer disease duration of patients with MOGAD.

Patients with RRMS showed bilateral thalamic atrophy compared to HC, as previously reported [25]. Thalamic atrophy is known to occur early in the disease course due to primary thalamic pathology as well as retrograde or anterograde neuraxonal degeneration after WM injury (e.g., demyelination) of tracts connecting the thalamus with other brain regions. No significant WM atrophy was demonstrated in patients with RRMS compared to HC in our study. Low lesion load and low clinical disability can be a reason for these results. On the other hand, inflammatory processes in early RRMS that lead to tissue edema can mask disease‐related WM atrophy [26]. No significant volume differences were detected between MS and MOGAD.

The primary aim of this exploratory study was to longitudinally investigate brain volume dynamics independent of relapse activity in two single‐center patient cohorts with MS and MOGAD as evidence of neurodegeneration. Of the 27 MOGAD patients included at baseline, 15 completed the 2‐year follow‐up imaging. Importantly, there were no significant differences in baseline demographic or clinical characteristics—including age, sex, EDSS, or lesion volume—between patients who completed follow‐up and those who did not, suggesting that the dropout is unlikely to have introduced systematic bias. As expected, we could demonstrate longitudinal WM and GM changes in the RRMS cohort. More specifically, progressive GM atrophy was detected in DGM structures (caudate nucleus), infratentorially (cerebellum) as well as in cerebral cortical areas in bilateral frontal lobes. Longitudinal WM atrophy involved WM structures adjacent to cortical GM in several frontal, temporal, and occipital gyri as well as in the cingulate gyrus (above corpus callosum). No longitudinal WM and GM changes have been observed in the MOGAD group. In order to examine the impact of disease activity on atrophy patterns, we repeated VBM including the occurrence of clinical relapses between baseline and follow‐up visits as a covariate in the analysis. Again, no longitudinal volume changes were shown for the MOGAD group. In our MS group, a part of VBM clusters of GM and WM remained significant, underpinning the importance of relapse—independent neurodegeneration, even in the early disease stages. Volume loss independent of relapse activity could be demonstrated in temporal WM and cerebellar as well as cortical GM regions in MS, compatible with the recent literature [6].

Two longitudinal volumetric studies have hitherto reported progressive total GM, thalamic, and hippocampal atrophy in MOGAD patients [27, 28]. The discrepancies between these findings and our results may be attributed to different demographics (smaller number of individuals, older patients with longer disease duration and higher lesion load), MRI data from scanners with different field strengths within a study, and different methodological approaches of postprocessing. In terms of demographics, both previous studies included MOGAD patients with a mean age of 41.7 and 39.2 years, respectively, while our cohort was younger (mean age 31 years), potentially reducing the impact of age‐related brain volume loss. Additionally, the higher lesion volumes reported in the studies by Lotan et al. and Amin et al. may have contributed to atrophy via Wallerian degeneration. Regarding the methodological differences between the aforementioned studies and our study, it is important to mention that VBM is less reliant on predefined tissue masks (used from segmentation‐based tools like SIENAX and icobrain ms) and enables voxel‐wise analysis of spatially localized changes. Furthermore, we strictly applied the 2023 diagnostic criteria on our MOGAD [15], avoiding false positive results in patients with MS as a more likely diagnosis [29].

We acknowledge the limitations of the present single‐center study. First, the exploratory nature of the study is linked to the small sample size of patients with early‐stage MOGAD, with a median follow‐up period of 2 years. The inclusion of only 15 MOGAD patients in the longitudinal analysis may increase the risk of a Type II error (i.e., false‐negative results). However, given the relatively low prevalence of MOGAD, it is inherently challenging to longitudinally follow a larger cohort of patients within a monocentric setting. Therefore, multicenter studies with standardized MRI protocols are needed to validate our results, to investigate differences in atrophy patterns in different clinical phenotypes, and to more effectively evaluate the influence of clinical relapses or lesion load on longitudinal tissue injury. Secondly, our focus was to examine the relapse‐independent volume changes; therefore, the observation in 4 patients who experienced a clinical relapse during the time between baseline and follow‐up visit is also purely explorative. Consequently, larger patient samples are needed to evaluate the effect of demyelinating lesions and relapse frequency on the longitudinal atrophy patterns. Thirdly, our results—especially—for WM atrophy should be compared with other advanced neuroimaging methods (e.g., diffusion tensor imaging).

In conclusion, the present study provides no evidence of disease progression reflected by ongoing brain atrophy in early MOGAD as opposed to early MS with low relapse frequency. No differences in regional GM and WM were detected over time in MOGAD, supporting the relapsing nature of the disease and contrasting it with RRMS and relapse‐independent progression. Moreover, WM atrophy in MOGAD compared to HC can presumably be explained by the history of ADEM or tumefactive lesions in the earlier course of the disease. Further prospective studies with a longer follow‐up time and larger cohorts are warranted to validate these findings.

## Conflicts of Interest

D.B., Z.A., A.K., B.K., B.B.: nothing to disclose. T.L.: has received research scientific grant support from Novartis Pharma. C.S. has received speaker honoraria from Alexion and travel support from Novartis and UCB. R.S. has received speaker's honoraria from Bayer HealthCare, Alexion Pharma, Novartis Pharma, and Roche Pharma AG, congress travel support from Merck, Biogen Idec GmbH, and has received research scientific grant support from Novartis Pharma. R.G.: has received compensation for serving as a consultant or speaker from Bayer HealthCare, Biogen Idec, Merck Serono, Novartis, and Teva Neuroscience; he, or the institution he works for, has received research support from Bayer HealthCare, Biogen Idec, Merck Serono, Novartis, and Teva Neuroscience; he has also received honoraria as a Journal Editor from SAGE and Thieme Verlag. I.A.: has received travel grants from Alexion, BMS, Horizon, Roche, Biogen Idec, and Guthy‐Jackson Charitable Foundation, served on scientific advisory boards for Merck, Roche, Alexion, Horizon, and Sanofi, and received research support from Diamed and Roche. C.L.: received a research grant from the German Federal Ministry for Education and Research, BMBF, German Competence Network Multiple Sclerosis (KKNMS), grant no. 01GI1601I, has received consulting and speaker's honoraria from Biogen Idec, Bayer Schering, Daiichi Sankyo, Merck Serono, Novartis, Sanofi, Genzyme, and TEVA.

## Supporting information


**Table S1:** Disease modifying therapies (DMDs) in the MS and MOGAD group at baseline.
**Figure S1:** Longitudinal VBM results for the MOGAD and the MS group including the occurrence of relapses between BS and FU. Statistically significant VBM cluster are marked with red/yellow for GM and blue/green for WM. All VBM results are at *p* < 0.05 with an extent threshold of > 50 voxels.

## Data Availability

The data that support the findings of this study are available on request from the corresponding author. The data are not publicly available due to privacy or ethical restrictions.
