# Supplementary material for: Longitudinal Brain Atrophy Patterns in Early MOG‐Antibody Associated Disease and Relapsing Multiple Sclerosis
Source: Eur J Neurol. 2025 Nov 18;32(11):e70354. doi: 10.1111/ene.70354 (PMC12627754; doi:10.1111/ene.70354)
Supplement: Supplementary file 1 — Table S1: Disease modifying therapies (DMDs) in the MS and MOGAD group at baseline. Figure S1: Longitudinal VBM results for the MOGAD and the MS group including the occurrence of relapses between BS and FU. Statistically significant VBM cluster are marked with red/yellow for GM and blue/green for WM. All VBM results are at p < 0.05 with an extent threshold of > 50 voxels. [file ENE-32-e70354-s001.docx]

**Supplementary material**

Supplementary Table 1. Disease modifying therapies (DMDs) in the MS and MOGAD group at baseline.

| **DMDs** | **MOGAD** | **RRMS** |
| --- | --- | --- |
| All patients | 27 | 40 |
| Patients on DMDs | 19 (70,4%) | 31 (77,5%) |
| Azathioprine | 7 (36,8%) | - |
| IVIG | 1 (5,3%) | - |
| Tocilizumab | 3 (15,8%) | - |
| Rituximab | 7 (36,8%) | 2 (6,4%) |
| IFN beta 1a/1b | - | 12 (38,7%) |
| Glatiramer acetate | 1 (5,3%) | 5 (16,1%) |
| Teriflunomide | - | 2 (6,4%) |
| Dimethyl fumarate | - | 5 (16,1%) |
| Fingolimod | - | 3 (9,7%) |
| Natalizumab | - | 2 (6,4%) |

**
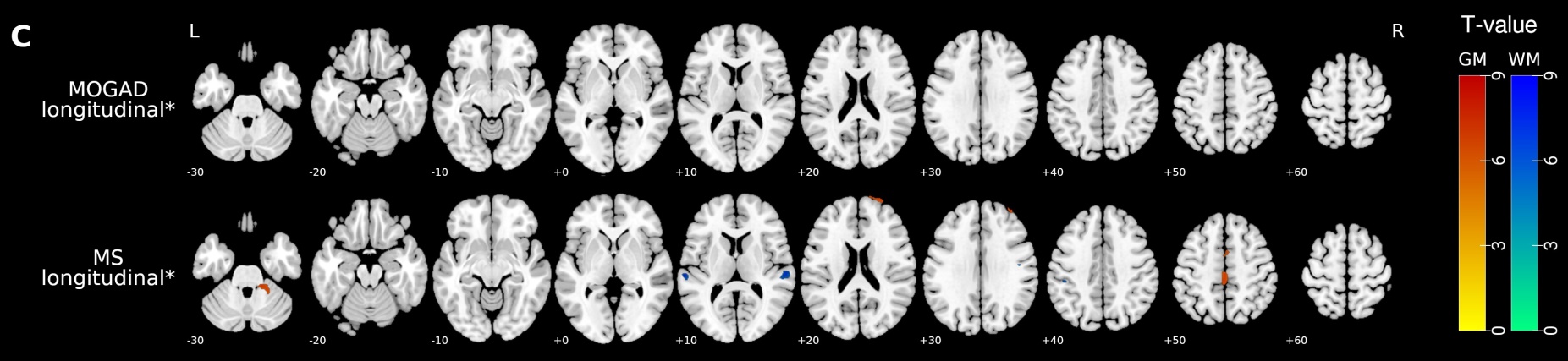
**

**Supplementary figure 1.** Longitudinal VBM results for the MOGAD and the MS group including the occurrence of relapses between BS and FU. Statistically significant VBM cluster are marked with red/yellow for GM and blue/green for WM. All VBM results are at p<0.05 with an extent threshold of >50 voxels.
